# Recent advancements in smart responsive tissue adhesives for medical applications

**DOI:** 10.3389/fmed.2025.1696667

**Published:** 2025-11-28

**Authors:** Shiqi Lin, Qiang Jin, Ji Wang, Sufan Wu, Yi Zhou

**Affiliations:** 1 Center for Plastic & Reconstructive Surgery, Department of Plastic & Reconstructive Surgery, Zhejiang Provincial People’s Hospital (Affiliated People’s Hospital), Hangzhou Medical College, Hangzhou, Zhejiang, China; 2 The Secondary School of Clinical Medicine College, Zhe Jiang Chinese Medical University, Hangzhou, China

**Keywords:** tissue adhesive, reversible, detachment, medical, mechanism

## Abstract

Bioadhesives have emerged as a promising alternative to conventional sutures, offering rapid and robust tissue integration while overcoming limitations such as time-consuming procedures and risks of secondary tissue damage. However, the bonding strength and degradation kinetics of most existing adhesives remain difficult to precisely control, limiting their adaptability to diverse tissue types and clinical scenarios. In this context, smart responsive bioadhesives have attracted significant attention due to their tunable adhesion properties, on-demand detachment capability, and tissue-matching mechanical properties. These materials can respond to biological environmental stimuli, enabling dynamic interactions with biological systems and opening new avenues for applications in wearable devices, controlled drug delivery, tissue repair, and smart bioelectronic interfaces. Nevertheless, a systematic review summarizing the synthesis strategies, adhesion mechanisms, and application prospects of such advanced adhesives remains lacking. Herein, we comprehensively analyzes the design strategies, working principles, and biomedical applications of smart responsive adhesives over the past decade. Furthermore, we discuss current limitations and propose future directions aimed at accelerating the translation of smart responsive adhesives from laboratory research to clinical practice. This review aims to provide a comprehensive understanding of these materials and offer valuable insights for researchers engaged in developing advanced tissue adhesives for practical applications.

## 1 Introduction

Adhesion is ubiquitous in nature. Octopuses and mussels anchor themselves to rocks, and remora fishes attach to other marine creatures, and geckos cling to walls (1–3). Inspired by these natural adhesion phenomenon, human beings have created various adhesives that can be applied to practical scenarios. The earliest adhesives in history could date back to the early days of humanity when people used water and clays to simply bind objects together. However, such adhesion methods were difficult to achieve long-lasting and strong bonding effects (4). As human society developed, more and more adhesives were gradually invented and began to gain recognition in human history. In particular, the discovery of polymer materials pushed the development of synthetic adhesives to a new height (5). These adhesives are now widely used in medicine, aerospace, machinery and other manufacturing industries.

For medical applications, compared to traditional techniques, medical tissue adhesives can achieve faster and more convenient tissue suturing and fixation (6). Additionally, due to their ease of use, tissue adhesives require less technical expertise from operators (7). Currently, medical tissue adhesives used in clinical practice are divided into natural adhesives and synthetic adhesives (8). Natural tissue adhesives cause less inflammation and foreign body reactions with higher biocompatibility, while they fail to achieve an ideal adhesion effect. Generally, natural adhesives can be divided into fibrin adhesives, gelatin-based adhesives, albumin-based adhesives, and polysaccharide-based adhesives (9). However, natural adhesives weaken with moisture and heat, exhibit inconsistent quality, and have limited bonding strength. These shortcomings drive the shift to synthetic alternatives, which offer superior durability, water resistance, and reliable performance in diverse conditions. In contrast, synthetic adhesives can provide strong and durable bonding effects, but their poor biocompatibility leads to severe inflammation and rejection reactions (10, 11). And synthetic tissue adhesives can be divided into polycyanoacrylate (PCA)-based adhesives, polyethylene glycol (PEG)-based adhesives, and polyurethane (PU)-based adhesives (10, 12).

Despite significant progress in the development of tissue adhesives, there are still some drawbacks that cannot be ignored. (1) The adhesion strength of adhesives is uncontrollable (13, 14). Although the adhesion strength of some tissue adhesives can ensure their bonding effect, the fault tolerance is low, resulting in secondary damage and bleeding to newly formed tissue during the detachment process, which has negative impacts on the ideal healing of the wound (15). (2) The adhesives do not match the elastic modulus of various of tissues (16). Tissue adhesives should ensure the normal physiological functions of tissues when bonding tissues, otherwise it will cause adverse consequences to organs and the human body itself, such as pulsation of the heart and blood vessels, and flexible movement at joints, respiratory function in the lungs (17). (3) The degradation rate of adhesives does not match the rate of tissue regeneration, making it difficult for granulation tissues to grow into damaged tissues and prolonging tissue healing and exposure time (18, 19). Wounds are more susceptible to get bacteria contamination and scar healing. Therefore, smart responsive tissue adhesives with advantages such as good biocompatibility, controllable adhesive strength, and a better match between degradation rate and tissue regeneration rate have been proposed (19, 20).

The development of highly biocompatible smart responsive tissue adhesives that allow for on-demand and non-invasive detachment has gradually become a current research focus. This review aims to introduce the reversible adhesion mechanisms of various smart responsive tissue adhesives, as well as the research progress and hotspots in biomedical applications.

## 2 Reversible adhesion mechanism

**FIGURE 1:**
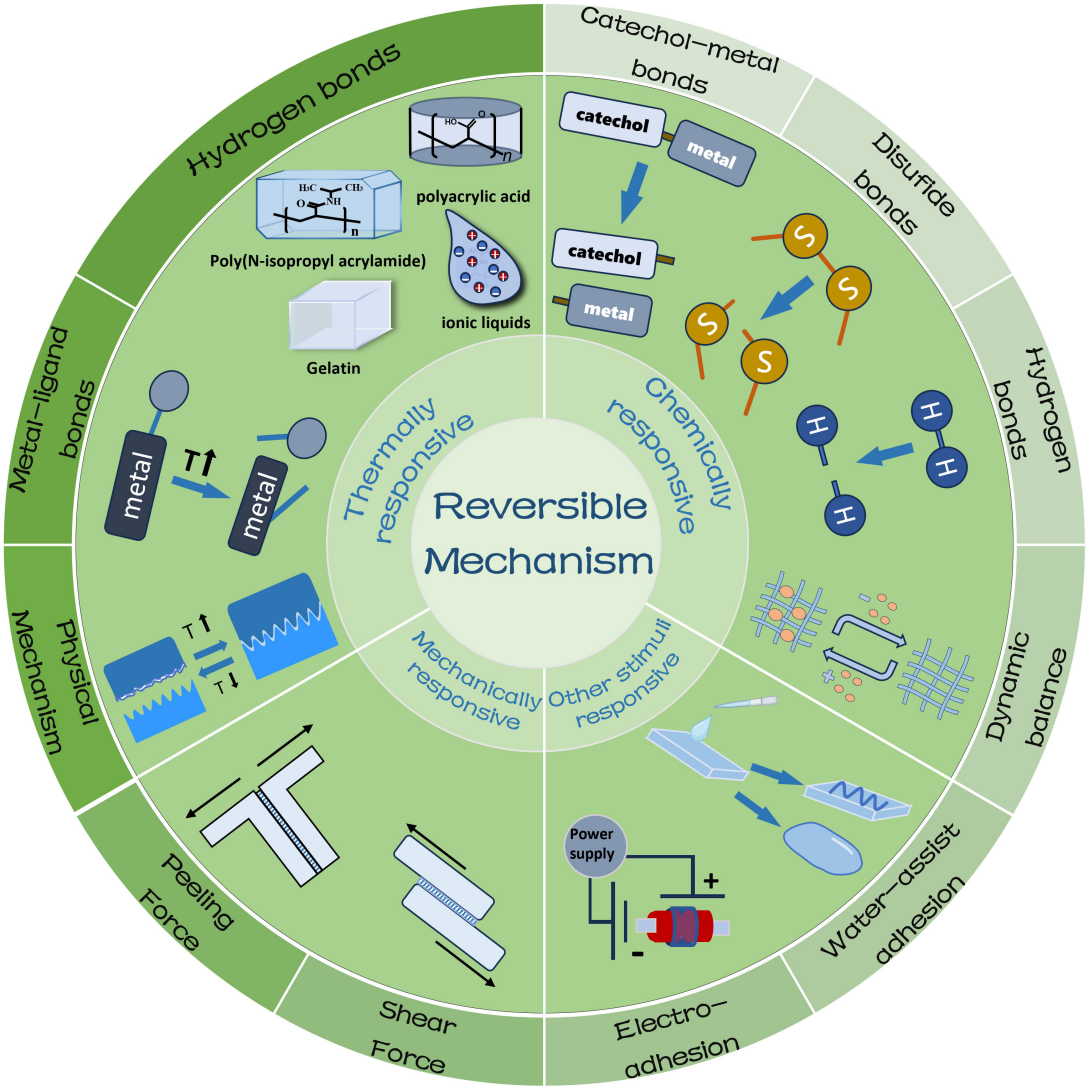
Schematic diagram of reversible adhesion mechanisms of smart responsive tissue adhesives.

**FIGURE 2:**
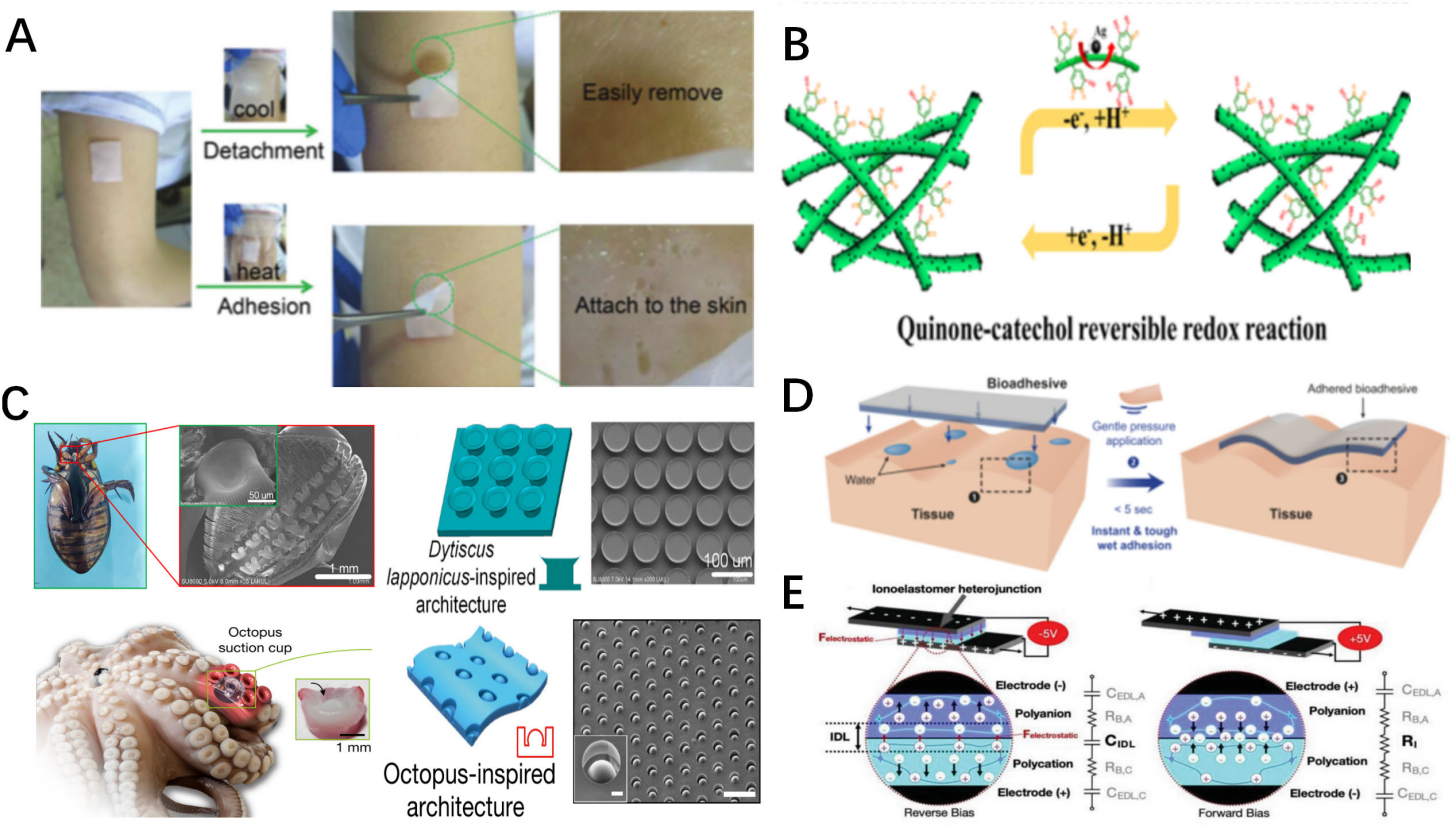
Schematic representation of various of responsive adhesives. **(A)**. Schematic representation of thermally responsive adhesion (38). **(B)** Schematic representation of dynamic balance between chemical functional groups of chemically responsive adhesion (111). As the balance is broke, the adhesion states change. **(C)** Schematic depiction of constructions of Dytiscus lapponicus-inspired adhesion structure (DIAS) and octopus-inspired architectures (OIAs) (65, 66). **(D)** Schematic representation of water-assist adhesion (57). **(E)** Schematic representation of and electro-adhesion (124).

Reversibility is a prominent advantage of smart responsive tissue adhesives, implying that the adhesive strength can be controlled for on-demand detachment (21). According to adhesion mechanisms, they can be categorized into four main types, namely thermally responsive (22–24), chemically responsive (25, 26), mechanically responsive (27) and other reversible mechanisms (28–30) (Figure 1). This section introduces the reversible adhesion mechanisms of the different types of smart responsive tissue adhesives (Figure 2) and their minimal and maximal adhesion strength are listed in Tables 1–4, respectively.

**TABLE 1:** Summary of reversible adhesion mechanism, maximal and minimal adhesion strength and biomedical applications of thermally responsive tissue adhesives.

Reversible adhesion mechanism		Adhesives	Substrate	Testing method	Adhesion		Response time	Peeling	Durability	Rupture strain	Biocompatibility	Application
					Maximum	Minimum						
Thermally response adhesives	Hydrogen bond	Poly(N-isopropylacrylamide)-Clay-polydopamine nanoparticles/poly(N-isopropylacrylamide)-Clay bilayer hydrogel (NC-PDA/NC (34))	Dry porcine skin	Tensile adhesion test	4.83 kpa (Room Temperature)	∼0 kpa (37 °C)	/	Without any residue	Remains 4.8 Kpa after 3 cycles of cyclic peel-off tests	850%	NIH-3T3	Wearable electronic devices
		Dynamic multiscale contact synergy hydrogel adhesive (DMCS) (35)	Dry glass/Wood	/	∼21 kpa/32 kpa (10–20 °C)	∼0.4 kpa/0.13 kpa (60 °C)	0.4 s	/	Reversibly switched about 50 times	Stretch to more than 7 times its original length	/	Intelligent robotics
		Poly(3,4-dihydroxy-L-phenylalanine)-adamantine -methoxyethyl acrylate-poly(N-iso-propylacrylamide-β-cyclodextrin (pDOPA-AD-MEA -pNIPAM-CD) (36)	Wet silicon substrate	Atomic Force Microscope (AFM)	∼23 nN (40 °C)	2.2 nN (25 °C)	/	/	Maintains a strong adhesion strength after 50 cycles	/	/	Intelligent robotics
		Octopus-inspired hydrogel adhesive (OHA) (37)	Dry porcine skin	/	∼0.3 mN (25 °C)	∼0.1 mN (45 °C)	/	Without secondary damage	Maintain highly robust adhesion after 1,000 attachment/detachment cycles	/	Human Umbilical Vein Endothelial Cells(HUVECs)	Target drug delivery system
		Chitosan–Catechol–poly (N-isopropyl acrylamide) (Chitosan–Catechol–pNIPAM) (38)	Dry Human arm	90° peeling test	0.038 N (40 °C)	∼0.0039 N (20 °C)	With NIR Laser for 30 s	Without any residue	/	/	Rabbit knee chondrocytes	Hemostatic adhesive materials
		2-(dimethylamino) ethyl metharcylate and N-isopropylacrylamide (DMAEMA/NIPAM) (39)	Dry glass	Peeling test	317.8 J/m2 (37 °C)	/	/	/	10 repeated peel tests	Stretching to 200% strain	/	Artificial skin sensing system
		Gelatin-sodium alginate- Tris/protocatechualdehyde/ferric chloride (GT-SA-TPF) (40)	Dry porcine skin	Lap shear test	38 kPa (37 °C)	11 kpa (25 °C)	few minutes	Peeled easily	Recover to original value after 5 temperature cycles	/	Murine fibroblast (L 929)/Rat	Acute wound dressings
		Quaternized chitosan-polyacrylic acid hydrogel (QAAH) (44)	Dry porcine skin	lap-shear adhesion	37.4 kpa (37 °C)	6.8 kpa (20 °C)	several seconds	Easy to detach	/	/	HUVECs	Artificial skin sensing system
		Quaternized chitosan@poly(1-vinyl-3-butyl imidazolium bromide)-co-methacryloyloxyethyl trimethylammonium chloride QCS/[BVIm][Br]/DMC (43)	Porcine skin/Glass	Tension-shear test	26.45 kpa/33.70 kPa (20 °C)	/	/	Easily removed	Remains above 71%/96% of the original after the cycles for 10 times	/	L929/Hemolysis performance	Multiple function wound dressing
		Polymerizing acrylamide (AM) monomer -polyethylenimine (PEI) -Glycerol-Ionic hybrid hydrogel (45)	Iron sheet	Tensile adhesion test	1.1 Mka (−40 °C)	102 Kpa (20 °C)			Do not attenuate for at least 5 cycles.	More than 300 times of its initial length	/	wearable electronic devices
	Dynamic metal-ligand bonds	Cu^2+^-curcumin-imidazole-polyurethane hydrogel adhesives (CIPUs: Cu^2+^) (48)	Aluminum sheet	Tensile adhesion test	2.46 Mpa (RT)	/	Exposed to the NIR light for 30 s	Loss of adhesion ability	CIPUs:Cu2 + can largely maintain after multiple repeated sticking/peeling operations	Maximum value of the tensile strength is 2.05 MPa	/	/
	Melting/crystallization	1-ethyl-3-methylimidazolium bromide crystal fiber ([EMIM]Br) (50)	Glass	Lap-shear test	3.47 kpa (20 °C)	0.016 kpa (60 °C)	Exposed to the NIR light (1 W) for 15 s	Without residue	remained the stable 3.31 ± 0.32 MPa after 10 cycles		/	Bioelectronics

**TABLE 2:** Summary of reversible adhesion mechanism, maximal and minimal adhesion strength and biomedical applications of chemically responsive adhesives.

Reversible adhesion mechanism		Adhesives	Substrate	Trigger	Testing method	Adhesion		Response time	Peeling	Durability	Rupture strain	Biocompatibility	Application
						Maximum	Minimum						
Chemically response adhesives	Catechol-metal bonds	Caffeic acid modified gelatin oxidized dextran-ZnO (CaGOD/ZnO) (54)	Porcine skin	Medical metal-chelating agent, ethylene diamine tetra-acetic acid (EDTA)	Lap shear test	26.9 kpa	4.3 kpa	/	Easily removed	/	Withstand the gravity of 100 g	NIH-3T3/hemolysis ratio	Multifunctional wound dressings
		Quaternized chitosan-ferric iron (protocatechualdehyde)_3_ QCS-PA@Fe (53)	Porcine skin	Medical metal-chelating agent, Deferoxamine mesylate (DFO)	Lap shear tests	10.4 kpa	1.3 kpa	/	Without secondary injury of the wound.	/	/	L929/mice	Multifunctional wound dressings
		Poly(acrylic acid-co-3-((8,11,13)-pentadeca-trienyl) benzene-1,2-diol –chitosan P(AA-co-UCAT)-CS (74)	Porcine skin	Metal solution (Fe3 +)/pH 8.0 aq. solution	180-degree peeling test	1664 J/m2	300 J/m^2^/752.4 J/m ^2^	/	Complete detachment	Maintains adhesion strength of 180 kPa after 20 adhering-peeling cycles	Highly stretchable (1,300–2,600%)	L929	Artificial skin sensors and wearable devices
	Disulfide bond	Aldehyde cellulose polyethylenimine -acrylic acid-3-sulfonic acid propyl methyl acrylic acid potassium - caffeic acid/polyethylene glycol diacrylate- carboxylated cellulose - acrylic acid CNC-CHO-PEI- AA- MASEP- CA/PEGDA- CNC-COOH- AA (CPAMC/PCA) (56)	Wet porcine myocardium tissue	Oxidized glutathione	Lap-shear tests	223.14 kpa	/	/	Quickly separate and remove	More than 30 cycles of attachment/detachmen	Did not get rupture after compressing to 60% strain	Cardiomyocytes	Multifunctional cardiac patch
		Polyvinyl alcohol-poly(acrylic acid) -N-hydroxysuccinimide (PVA-PAA-NHS) (57)	Wet por- cine skin	Solution consisting of sodium bicarbonate (SBC) and glutathione (GSH)	180° peel test	400J/m^2^	/	5 minutes	Nearly complete detachment	/	/	Sprague–Dawley rats	Removable implantable devices
	Hydrogen bonds	Tannin–europium coordination complex -citrate-based mussel-inspired bio-adhesives (TE-CMBA) (61)	Wet porcine skin	Borax solution	Lap shear strength tests	38.5 kpa	/	1.5–15 min	Leaving nearly no residue	Fully restored after four cycles.	The tensile strengths were > 400 kPa	HUVECs/L929 cells	Diabetic wound dressings
		Polyethylene glycol diacrylate/tannic acid (PEGDA/TA hydrogels) (62)	Underwater p orcine skin	Dimethyl-sulfoxide (DMSO) or urea	Tensile pull-off test	158.4 kpa	<5 kpa	15 s	/	20 cycles of adhesion tests	fracture energy:17.9 ± 2.1 kJ/m2	NIH-3T3/Sprague Dawley rats	Multifunctional wound dressings
		PEDOT: PSS @poly(vinyl alcohol)/poly(acrylic acid) (PEDOT: PSS PVA/PAA) (98)	Porcine skin	/	Lap shear test	15.9 kPa	/			Remained almost the same over 20 cycles	6 times of its original length	/	Wearable soft electronic devices
	Functional groups balance	Phenylboronic acid/Zn-CuO@ Graphite oxide nanosheets biofoam adhesive (PBA/Zn-CuO@GO) (63)	Human skin	Hydroxy-rich glucose solutions	/	Effectively attach	Dissociate and detachment	/	Easily removed	/	/	Human lung cancer cells (A549)	Multifunctional wound dressings
		Ag/Tannic acid -Cellulose nanofibers (Ag/TA-CNF) (111)	Porcine skin	/	Tensile adhesion tests	36.2 kPa	/	/	Without residual and damage	20 adhering peeling cycles	Maximum fracture energy of 2200 J/m2	L929 cells	Target drug delivery system

**TABLE 3:** Summary of reversible adhesion mechanism, maximal and minimal adhesion strength and biomedical applications of mechanically responsive tissue adhesives.

Reversible adhesion mechanisms		Adhesive	Substrate	Trigger	Testing method	Adhesion		Response time	Peeling	Durability	Rupture strain	Biocompatibility	Application
						Maximum	Minimum						
Mechanically Responsive Adhesion	Peeling-off force	D. lapponicus-inspired adhesion structure (DIAS) (65)	Dry/underwater glass dry/underwater porcine skin	Peeling-off force	Lap shear test	205/133 kpa ∼14 kpa/∼2 kpa	/	/	No residue	60 repeating cycles	/	/	Electronic skin-attachable devices
		Octopus-inspired architectures (OIAs) (66)	Wet/underwater/oil Silicon sheet on porcine skin	Peeling-off force	Adhesion Tester, Neo-Plus	37/41/154 kPa ∼25 kPa;	/	/	/	/	/	/	Applied over skin or wounds
		Soft-rigid hybrid smart artificial muscle (SRH-SAM) (67)	Dry glass	Peeling-off force	Custom-made apparatus	1.65N	0.88 N	/	/	/	Maximum contraction strain of 40%	/	Artificial muscles of soft grippers.
	Shear force	Alginate and polyacrylamide- N-fluorenylmethoxy-carbonyl-L-tryptophan (Alg/PAM-FT) (68)	Human skin	Peeling-off force	180° peel test	72 J/m^2^	20 J/m^2^	/	No residue	/	/	/	Pharmaceutical and electronic adhesives

**TABLE 4:** Summary of reversible adhesion mechanism, maximal and minimal adhesion strength and biomedical applications other stimuli responsive tissue adhesives.

Reversible adhesion mechanism		Adhesives	Substrate	Trigger	Testing method	Adhesion		Response time	Peeling	Durability	Rupture strain	Biocompatibility	Application
						Maximum	Minimum						
Other Stimuli-responsive adhesion	Water-assisted reversible adhesion	Polyvinyl alcohol/boric acids film (PVA/BA) (71)	Glass/transcutane	Water	Transcutaneous tensile test	570 N/cm2	49 N/cm2	30 s/dissolved in water for 2 hours	Easily peeled off	Adhesive strength increased back to 330 N/cm2 again	(> 200% strain	/	Wound dressing adhesives
		Polyvinyl alcohol/borax/oligomeric procyanidin hydrogels (PBO) (72)	Porcine skin		Lap shear test	29.2 kpa	/	10 min	Rapidly degrade	/	Highest storage modulus (G’): 3722.1 Pa	L929/mice	Smart hemostatic material
		Poly (acrylic acid-co-acrylamide) [poly (AA-co-AM)] (101)	Porcine skin		Shear adhesion tests	61.7 ka	/	/	Discharged automatically			Fibro-blast cells (FBs)/HUVECs	Skin flap transplantation
	Electroadhesion	Quaternized dimethyl aminoethyl methacrylate gel (QDM gel) (76)	Bovine tissue		Lap-shear test	20 kpa	/	/	Be peeled off intact	/	Same as the adhesion strength		Multifunctional wound dressings

### 2.1 Thermally responsive adhesion

Thermally responsive adhesives are the most common type, which are synthesized by mixing thermal-sensitive materials with adhesive materials (31). In such adhesives, the temperature is controlled to alter the state of chemical bonds or the adhesion state, thereby modulating the adhesive strength (22).

Reversible hydrogen bonds formed by thermosensitive materials and water molecules confer thermally responsive characteristics. Poly(N-isopropyl acrylamide) (pNIPAM) is an ideal thermosensitive material whose amide groups form hydrogen bonds with water molecules, increasing the hydrophilicity of adhesives at low temperatures. These hydrogen bonds break at high temperatures, turning the adhesives hydrophobic (32, 33). Based on the above mechanism, adhesives containing pNIPAM achieve temperature-controlled adhesion through four mechanisms. (1) Temperature-Controlled Exposure of Adhesive Functional Groups: Di and Zhang et al. developed pNIPAM-Clay-polydopamine nanoparticles/pNIPAM-Clay (NC-PDA/NC) bilayer hydrogel adhesives and dynamic multiscale contact synergy (DMCS) hydrogel adhesives, respectively (34, 35). At room temperature, the pNIPAM inside the adhesives becomes hydrophilic, increasing the exposure of hydrophobic adhesive groups, thereby leading to enhanced adhesion. As the temperature rises, the hydrogen bonds break, resulting in reduced exposure of the adhesive functional groups, leading to decreased adhesive strength. Conversely, Zhao et al. synthesized a wet adhesive composed of guest copolymer 3,4-dihydroxy-L-phenylalanine (DOPA)-polymer-adamantine (AD)-methoxyethyl acrylate (MEA) and host copolymer pNIPAM-β-cyclodextrin (pNIPAM-CD), which absorbs water and swells at low temperatures (36). This effectively restricts the hydrophilic adhesive groups, thereby lowering the adhesive strength. As the temperature rises, the adhesive strength is enhanced due to the increased exposure of adhesive groups. (2) Temperature-Controlled Effective Bonding Area: Lee et al. designed an octopus-inspired hydrogel adhesive (OHA) that absorbs water and swells at low temperatures, thus increasing the effective bonding area and adhesion strength (37). As the temperature rises, the adhesive area decreases, leading to reduced adhesive strength. (3) Temperature-Controlled Sol-Gel State Change: Xu et al. prepared a Chitosan-Catechol-pNIPAM adhesive that absorbs water and remains in a sol state at low temperatures, exhibiting low friction and adhesive strength. However, the adhesive dehydrates and gels at high temperatures, increasing the friction and adhesion strength (38). (4) Temperature controlled effective bonding force strength. Sun et al. proposed the 2-(Dimethylamino)ethyl methacrylate/PNIPAM hydrogel (PDMAEMA/PNIPAM), which forms electrostatic complexations with tissues to achieve adhesion (39). However, at low temperatures, the electrostatic complexations fail and the adhesive can be removed easily.

Other thermosensitive materials can also be used to confer thermally responsive properties through hydrogen bonds. Using the temperature phase transition characteristic of gelatin (GT), Liang et al. synthesized a thermally responsive GT-sodium alginate (SA)-TPF (a compound composed of Tris, PA, and Fe^3+^) hydrogel adhesive, named GT-SA-TPF (40–42). In this case, GT-related hydrogen bonds break at high temperatures, decreasing the cohesiveness of the adhesive and increasing the exposure of adhesive groups and adhesion. At low temperatures, the exposure of adhesive groups decreases, leading to decreased adhesion. Hydrogen bonds formed by ionic liquids (ILs) within the adhesives’ networks break at high temperatures, promoting hydrophobic interactions and increasing adhesive strength, whereas the adhesive strength decreases at lower temperatures. Zhang et al. created an IL-based hydrogel adhesive, quaternized chitosan (QCS)@poly(ILs)-co-methacryloyloxyethyl trimethylammonium chloride, QCS@P(IL-co-DMC), with temperature-controlled adhesion (43). Additionally, polyacrylic acid (PAA) releases a large number of carboxyl groups upon heating, which interact with other groups to form hydrogen bonds, thereby increasing adhesive strength. Shi and Wu utilized this mechanism to design QCS-polyacrylic acid hydrogel (QAAH), where the adhesion mechanism weakens or even disappears in low-temperature environments, enabling reversible adhesion (44). Yan et al. designed a low-temperature resistant glycerol-ionic hybrid hydrogel with reversible adhesion (45). At low temperatures, the hydrogen bonds, interface interactions, and tensile strength simultaneously increase and collaboratively enhance the adhesive strength. Therefore, the adhesive strength at −40°C is significantly higher than that at room or higher temperatures.

Moreover, dynamic metal-ligand bonds also provide thermally responsive properties to adhesives (46, 47). Xu et al. introduced Cu ions to prepare Cu^2+^-curcumin-imidazole-polyurethane hydrogel adhesives (CIPUs: Cu^2+^), which achieve high adhesion by interacting with tissues through Cu-ligand bonds (48). The metal-ligand bonds break under near infrared (NIR) assisted photothermal effects, resulting in reduced adhesion; however, the metal-ligand bonds reform at room temperature, restoring the adhesion.

Furthermore, thermally-responsive adhesives can achieve reversible adhesion through physical mechanisms (2, 49). Xi et al. developed a 1-ethyl-3-methylimidazolium bromide ([EMIM]Br) crystal fiber-reinforced polymer gel, EBrH-GEL, based on crystal melting/crystallization mechanisms (50). The crystals within the EBrH-GEL melt at high temperatures and adhere to the substrates tightly, which then *in situ* crystallize at low temperatures, anchoring to the tissues and forming strong adhesion.

In summary, temperature changes can dynamically control the adhesion strength of thermally responsive adhesives through various mechanisms, including phase transitions, reversible hydrogen bonding, and metal coordination bonding.

### 2.2 Chemically responsive adhesion

Chemically responsive adhesives achieve controllable adhesive strength by disrupting the chemical bonds within adhesives or between adhesives and tissues. These chemical bonds mainly include catechol-metal bonds, redox-sensitive disulfide bonds, and hydrogen bonds (51).

Introducing catechol-metal bonds into adhesives can enhance their adhesion (52) Cheng et al. and Liang et al. developed hydrogel adhesives with strong tissue adhesion. One is composed of caffeic acid-modified gelatin (CaG), oxidized dextran (ODex), and ZnO (referred to as CaGOD/ZnO), and contains catechol-Zn bonds; the other is composed of quaternized chitosan-Ferri iron (protocatechualdehyde)_3_ (PA@Fe) (QCS-PA@Fe), containing catechol-Fe bonds (53, 54). Medical metal chelators, such as: ethylene diamine tetra-acetic acid (EDTA) and deferoxamine mesylate (DFO), can disrupt these catechol-metal bonds to reduce their adhesive strength. Ultimately, chemically responsive adhesives can be removed by minimally invasive methods.

In addition, disulfide bonds can be disrupted by redox reactions (55). Researchers have introduced disulfide bonds into adhesives in various ways to prepare redox-responsive adhesives. He et al. introduced disulfide bonds through crosslinkers to create a CPAMC/PCA Janus hydrogel (56). Chen and Yu et al. prepared polyvinyl alcohol-poly(acrylic acid)-N-hydroxysuccinimide (PVA-PAA-NHS) by forming disulfide bonds through the reaction of -NHS ester and PAA (57). These adhesives can adhere tightly to various tissues but can also be easily removed with glutathione, which disrupts the disulfide bonds in adhesives (58).

Reversible hydrogen bonds formed between catechol groups and tissues could be replaced by interference from other groups (59). Fu et al. designed tannin–europium coordination complex crosslinked citrate-based mussel-inspired bio-adhesives (TE-CMBA), in which the catechol-hydrogen bonds were replaced by boric acid ester bonds after treatment with borax solutions, making the adhesives soluble and removable (60, 61). Similarly, Chen produced polyethylene glycol diacrylate/tannic acid (PEGDA/TA) hydrogels in which the catechol-hydrogen bonds were replaced after treatment with dimethyl sulfoxide (DMSO) or urea, resulting in reduced adhesion (62). Upon tissue healing, the adhesive can be on-demand removed by adding DMSO or urea to prevent secondary damage during separation.

Some chemically responsive adhesives maintain adhesion by sustaining a dynamic balance between functional groups, such as the phenylboronic acid (PBA)/Zn-CuO@GO nanosheet biofoam adhesive (PBA/Zn-CuO@GO) developed by Liu et al. (63). The adhesive takes advantage of the dynamic balance of phenylboronic acid/cis-diol interactions to sustain its adhesive property. When the balance is disrupted by competitive inhibitors (hydroxy-rich glucose solution), the adhesive strength decreases, leading to dissolution and detachment (64).

Chemically responsive adhesives typically achieve controlled debonding by introducing easily breakable chemical bonds in the cross-linked network. When these chemical bonds are broken, the cross-linking network collapses, and the adhesive becomes inactive and detached.

### 2.3 Mechanically responsive adhesion

As opposed to the aforementioned adhesive mechanisms, mechanical adhesives do not rely on environmental changes or additional reagents. Instead, they leverage the unique properties of materials or structures to achieve reversible adhesion (27).

Inspired by natural adhesive structures, researchers have developed bio-inspired adhesives that can be detached using peeling forces. Li et al. created a Dytiscus lapponicus-inspired adhesion structure (DIAS) that mimics the adhesive setae of Dytiscus lapponicus and can be removed with peeling force (65). Furthermore, the octopus-inspired architectures (OIAs) designed by Baik et al. are also structural adhesives relying on peeling force for detachment (66). These adhere to tissues through suction generated by structural deformations, which are easily broken by peeling force.

Zhao and Tian et al. designed a soft gripper consisting of an adhesive film and artificial muscles, soft-rigid hybrid smart artificial muscle. In the absence of electricity, the muscles remain relaxed and the adhesive film layer generates high adhesion strength through van der Waals forces and capillary forces. However, when the gripper is electrified and the artificial muscles contract, the edges of the film deform and break the adhesion mechanism, resulting in the detachment of the adhesive (67).

N-Fluorenylmethoxy-carbonyl-L-tryptophan (FT) is prone to disassembly under shear force. Wang et al. incorporated FT into the hydrogel network of alginate (Alg) and polyacrylamide (PAM) to synthesize the Alg/PAM-FT hydrogel (68). The networks within the hydrogel break under external shear forces, and the adhesion fails.

The mechanical force-responsive adhesives can achieve debonding without external stimulation or causing additional damage to adjacent tissues and objects. This capability demonstrates potential for practical applications and clinical translation.

### 2.4 Other stimuli-responsive adhesions

In addition, researchers have proposed other types of responsive adhesives, such as water-assisted adhesives active by moisture, and electrically responsive adhesives triggered by specific current directions; both represent good innovations.

Water-assisted reversible adhesion mechanisms include two types. (1) Water-dissociable chemical bonds, such as boronic ester bonds: When water-assisted reversible adhesives contact with a small amount of water, the boronic ester bonds within adhesives dissociate partially and rebond with tissues (69, 70). When immersed in excess water, the bonds in adhesives completely break, reducing the adhesion. Both the polyvinyl alcohol/boric acids (PVA/BA) film reported by Chen et al. and the PBA/borax/oligomeric procyanidin (OPC) hydrogels (PBO) reported by He et al. contain reversible boronic ester bonds and exhibit the above-mentioned reversible adhesion (71, 72). (2) The second mechanism employs water absorption to adjust the amount of effective adhesive bonds and the contact area (73). Using acrylic acid (AA) and acrylamide (AM), Zhou et al. designed poly(AA-co-AM) hydrogel adhesive, which wrinkles and softens when absorbing moisture from wet tissue to increase the effective adhesive area and the amount of hydrogen bonds, thereby enhancing adhesion. When exposed to excess water, wrinkles flatten or even disappear, lowering the adhesion (74).

Furthermore, electro-adhesion represents another reversible adhesion mechanism, in which two substances with opposite electrodes adhere directionally under the effects of electricity (75). The exact mechanism remains unclear but may result from molecular rearrangement under the influence of electrical fields (76). Broden et al. designed a positively charged quaternized dimethyl aminoethyl methacrylate gel (QDM) that can generate an adhesion strength of 25 kPa on bovine aorta when a specific directional current is applied. Reversing the electric field immediately reduces the adhesion strength to 5 kPa, allowing for rapid, non-invasive detachment (76).

**FIGURE 3:**
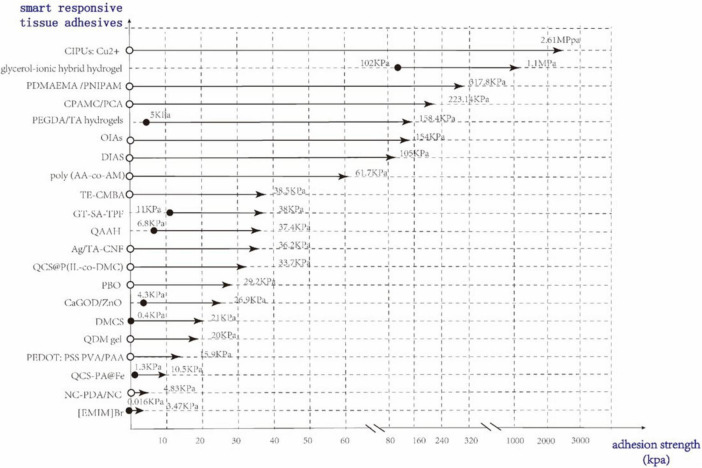
Part of smart responsive tissue adhesives’ minimal and maximal adhesion strength.

In a word, the smart responsive mechanisms mentioned above are currently the focus of research, and the smart responsive adhesives mentioned above can effectively achieve reversible adhesion effects. Their relevant information can be seen in Tables 1–4, and parts of smart responsive tissue adhesives’ minimal and maximal adhesion strength are shown in Figure 3.

## 3 Biomedical applications of smart responsive tissue adhesives

**FIGURE 4:**
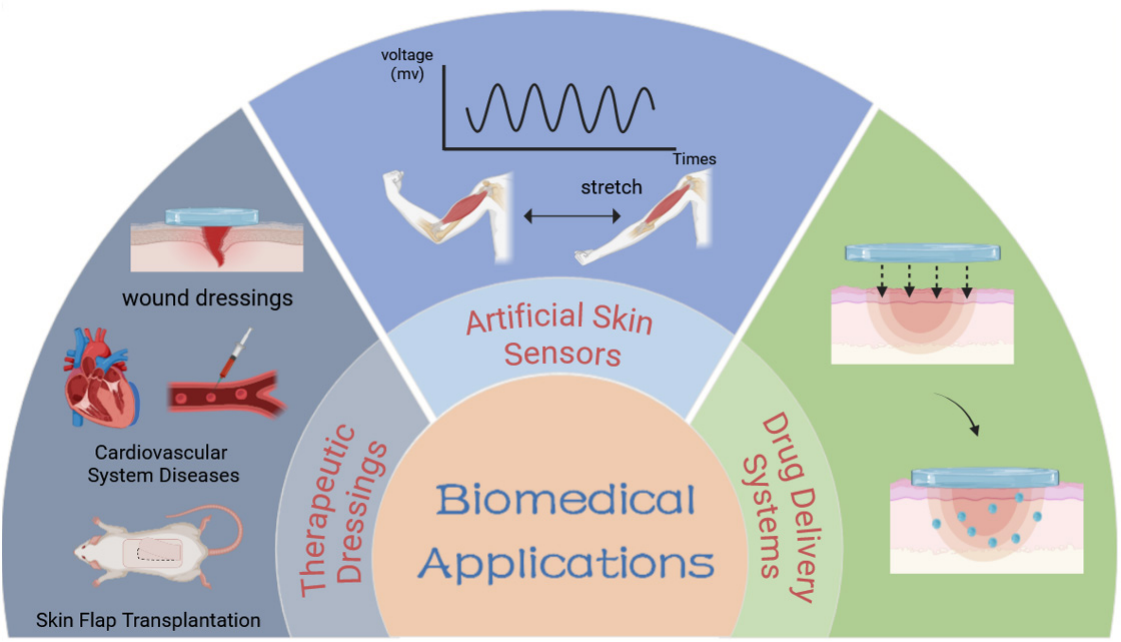
Schematic diagram of biomedical applications of smart responsive tissue adhesives.

**FIGURE 5:**
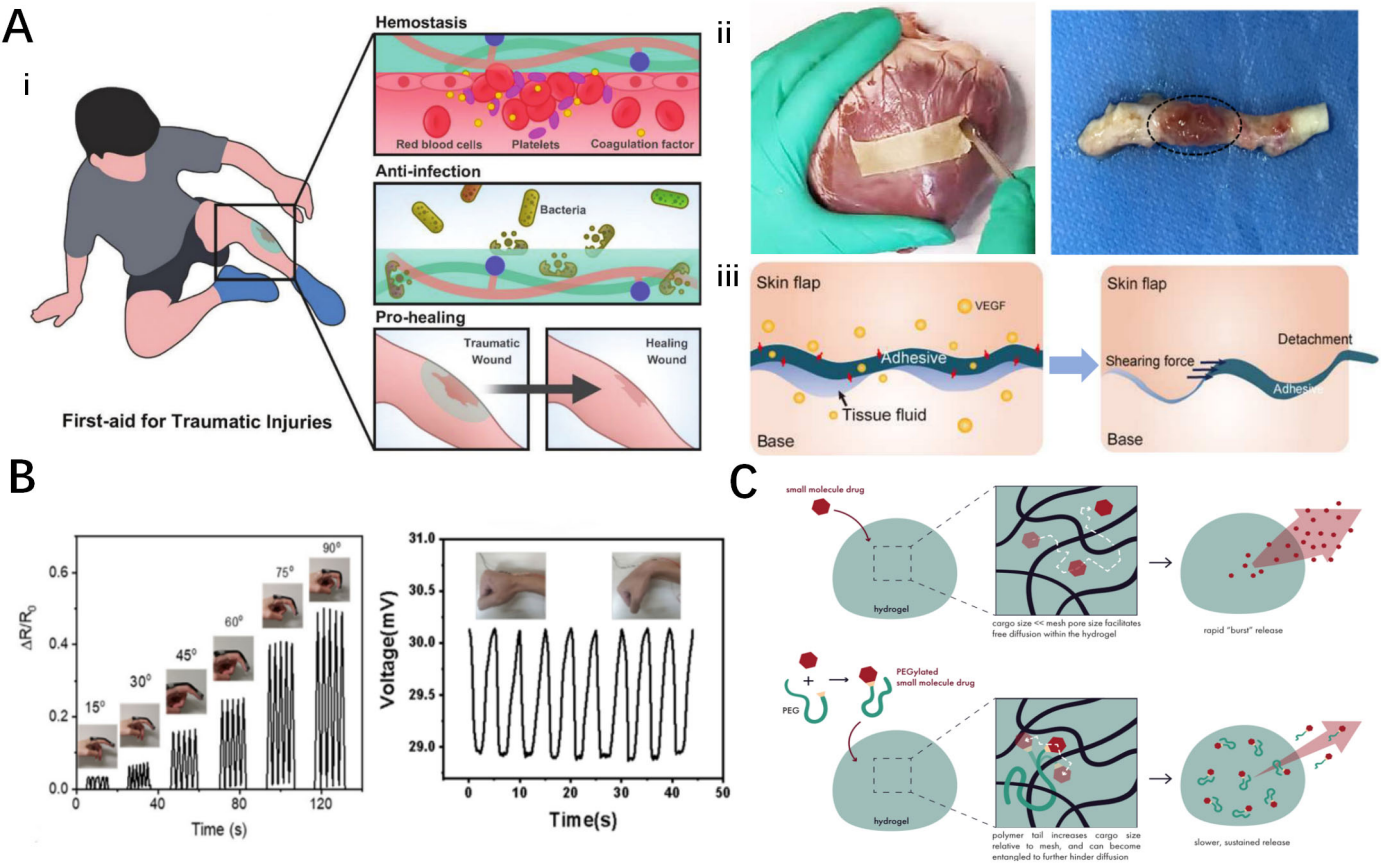
Biomedical applications of smart responsive tissue adhesives. **(A)** Smart responsive tissue adhesives for therapeutic dressings: (i) wound dressings for acute injuries (54), (ii) dressings for cardiovascular system diseases (56, 94), (iii) dressings for skin flap transplantation (101). **(B)** Smart responsive tissue adhesives for artificial skin sensors (39, 98). **(C)** Smart responsive tissue adhesives for drug delivery system (125).

Cyanoacrylates (e.g., Epiglu^®^, Dermabond^®^) and fibrin sealants are clinically approved and extensively utilized, showing superior efficacy compared to traditional suturing in multiple clinical trials, however, they lack smart responsiveness. Consequently, the development of intelligent adhesives with controllable adhesion properties holds significant clinical potential. To achieve reversible adhesion, non-invasive detachment, and residue-free removal, smart responsive tissue adhesives have been increasingly studied in the biomedical fields. Currently, the primary applications of smart responsive tissue adhesives include tissue adhesives (9), artificial skin sensors (77), and drug delivery systems (78). This section briefly introduces the research progress in these biomedical fields (Figures 4, 5).

### 3.1 Therapeutic dressings

Tissue adhesives can be applied in multiple clinical scenarios, such as wound dressings, cardiovascular system diseases and surgical incisions, and skin flap transplantations. Damaged tissues in direct contact with the external environment may lead to serious complications if they are not treated promptly and effectively. Therefore, the development of multifunctional therapeutic dressings holds significance (79, 80).

#### 3.1.1 Wound dressings

Acute wounds are often accompanied by significant bleeding and bacterial contamination, while chronic wounds are typically in a state of prolonged inflammation and slow healing (9, 81). Conventional gauze used in clinical practice has limitations such as poor hemostatic effect, lack of antimicrobial effect, and the potential to damage newly formed tissue upon removal (82). Multifunctional smart wound dressings can address these deficiencies.

The wound bed after damage is susceptible to bacterial contamination, highlighting the importance of antibacterial properties in adhesive dressings (83). A widely adopted strategy involves incorporating antibacterial materials into the adhesives, such as nanoparticles bearing antibacterial properties. Liu et al. have developed the PBA/ZnCuO@GO biofoam based on Zn-CuO@GO nanosheets, which can kill 98% of Escherichia coli (*E. coli*) (63). Another antibacterial strategy involves using photothermal conversion materials to locally generate heat and denature the enzymes, thereby decreasing bacterial viability (84). In addition, Liang et al. incorporated iron ions into quaternized chitosan to prepare QCS-PA@Fe hydrogel adhesives with controllable NIR-assisted photothermal effect, which can kill bacteria without damaging normal tissues (53).

The hemostatic performance of medical adhesives plays a crucial role in acute bleeding wounds (79, 85). He et al. synthesized a rapidly hemostatic dressing with porous structures, named PBO hydrogel. This adhesive can effectively seal wounds and promote the aggregation of blood cells and the formation of blood clots to reduce bleeding time and volume (72).

Proper inflammation promotes the orderly healing of wounds, whereas excessive inflammation stimulates scar formation (86, 87). The phenolic groups in TA inhibit reactive oxygen species and free radicals to prevent excessive inflammation in the early stages of wound healing (88, 89). Concurrently, Chen et al. developed anti-inflammatory PEGDA/TA hydrogels. Wounds treated with this adhesive exhibited milder inflammation, faster healing, richer fibroblasts, and more orderly wound healing (62).

In chronic and difficult-to-heal wounds, wound dressings that promote angiogenesis can accelerate healing (90, 91). Lanthanide ion europium has been found to promote neovascularization. For this purpose, Fu et al. proposed tannin–europium coordination complex crosslinked citrate-based mussel-inspired bio-adhesives (TE-CMBAs) containing Eu-phenol ligands. In this adhesive, Eu ions promote the formation of new blood vessels, granulation tissue, and richer skin appendages, combined with the antioxidant and anti-inflammatory properties of TA (61).

The aforementioned properties are vital for wound adhesive dressings. Hence, the development of all-in-one multifunctional wound adhesive dressings has become a major research focus (92). Fortunately, the wound adhesives currently under development not only simultaneously address the above-mentioned properties but also maintain their respective characteristics, thereby presenting broad application prospects (93).

#### 3.1.2 Cardiovascular system diseases

The cardiovascular system plays a crucial role in maintaining circulation. Severe cardiovascular diseases typically have poor prognosis; in recent years, tissue adhesives have introduced new strategies for treating these conditions (94).

Trauma is one of the most common sources of peripheral vascular injuries (95). Vascular sealants with excellent hemostatic properties have promising applications. Liang et al. designed GT-SA-TPF, and Borden et al. developed an electrically-triggered QDM gel (40, 96). Both of them can adhere tightly to animal vascular tissues and withstand burst pressures higher than normal human blood pressure, making them ideal materials for sealing damaged vessels. Peripheral vascular damage can also be caused by injections. Xu et al. proposed a temperature-controlled chitosan-catechol-pNIPAM hydrogel coating for syringes (38). Mouse femoral vein models showed that it could effectively seal post-injection vessels and reduce bleeding.

Cardiovascular injuries usually result in significant bleeding and high mortality rates, while timely and effective hemostatic measures can improve the prognosis of severely injured patients (97, 98). Cheng et al. created the CaGOD/ZnO hydrogel by incorporating zinc ions to accelerate coagulation. Mouse cardiac perforation bleeding models demonstrated that the CaGOD/ZnO hydrogel not only reduced bleeding time and volume but also adhered stably to the ventricular wall, improving survival rates in cardiovascular injuries (54).

Myocardial ischemia (MI) and infarction are common cardiovascular diseases where inflammation induced by dying myocardial cells worsens the prognosis (99, 100). To address this issue, He et al. proposed the CPAMC/PCA Janus hydrogel cardiac patch (56). Containing ion-promoting agents, the CPAMC/PCA Janus hydrogel exhibits good electrical conductivity which could promote the maturation and functional recovery of myocardial cells. The patch contains large amounts of antioxidant and anti-inflammatory groups, effectively removing free radicals at the infarction site and reducing local inflammation. Additionally, echocardiography and histological analysis were performed in mouse heart attack models, revealing that the patch promotes neovascularization and structural and functional recovery of the myocardium, thereby improving the prognosis of MI (56).

#### 3.1.3 Skin flap transplantation

Flap transplantation is a common clinical surgery, but flap survival rates remain suboptimal. Zhou et al. designed a pattern-tunable wrinkle hydrogel film adhesive to stabilize graft flaps and inhibit inflammation, while stimulating angiogenesis, thus improving graft flap survival (101).

### 3.2 Artificial skin sensors

Skin is a multifunctional organ that receives various stimuli from the external environment and provides corresponding feedback to the brain. Inspired by the human skin, the concept of artificial skin sensors has been proposed (102). They can sensitively monitor human biological signals, such as blood pressure, heart rates, and temperature to assess the overall health condition (103).

Artificial skin sensors achieve conductivity by introducing ions or ion channels into hydrogels to alter their electrical conductivity (104, 105). Di et al. developed a thermosensitive reversible nanocomposite hydrogel adhesive (NC-PDA/NC) containing a large number of freely mobile ions (34). This hydrogel is highly responsive to changes in compression and stretching states, leading to alterations in its resistance and conductivity. Additionally, some materials can form ion channels after ion binding. For instance, the QAAH hydrogel prepared by Shi et al. contains many ion channels and exhibits excellent electrical conductivity, enabling the detection of both temperature and pressure; the resulting changes in resistance achieve signal perception and transmission (44).

Introducing conductive polymers could also endow hydrogel adhesives with signal sensing and conduction capabilities (106, 107). Peng et al. incorporated the conductive polymer PEDOT: PSS into a double network hydrogel of poly(vinyl alcohol)/poly(acrylic acid) (PVA/PAA) to prepare PEDOT: PSS@PVA/PAA hydrogel (98). Under different tensile strains, the resistance of the PEDOT: PSS@PVA/PAA hydrogel changes responsively. This hydrogel has been tested to detect small human activities, such as wrist pulses and neck vocal cord vibrations, and has yielded satisfactory results (98).

Furthermore, Sun et al. proposed a special self-powered gradient hydrogel that showed strong adhesion with tissues via hydrophobic interactions (39). The self-induced electric potential changes with its thickness. Specifically, when the thickness of the hydrogel is altered by external pressure, the internal ion concentrations and the maximum output voltages of the hydrogel also change. This hydrogel can detect external signals, provide sensitive signal feedback, and return to its initial state rapidly when the pressure is released (39).

### 3.3 Drug delivery systems

Targeted drug delivery systems ensure that drugs reach therapeutic sites accurately, while controlled-release systems sustain local drug concentrations (108). Both types of drug delivery systems can improve therapeutic efficiency, reduce treatment costs, and minimize wastage. However, achieving on-demand regulation of drug delivery system duration remains a challenge.

Innovative micro drug delivery systems aim to release drugs at the targeted sites via minimally invasive debonding under external interventions (109). Lee et al. designed a temperature-sensitive adhesive inspired by octopus suckers, known as octopus-inspired hydrogel adhesive (OHA), which is applied as the base of microrobots (37). These microrobots can adhere tightly to the targeted sites and detach and exit from the bodies with minimal invasiveness after treatments under human interventions.

Controlled drug release can be achieved by introducing materials with drug dispersion advantages into highly biocompatible intelligent hydrogel adhesives (110) Chen et al. introduced electrospun polyurethane nanofibers (NF) into a hydrogel composed of Ag/TA -Cellulose nanofibers (Ag/TA-CNF) to prepare a bilayer nanocomposite hydrogel (NF@HG) (111). This adhesive adheres tightly to tissues through catecholamine chemistry and releases drugs slowly and sustainably *in situ*, thereby enhancing local drug concentrations.

## 4 Research hotspots and challenges

**FIGURE 6:**
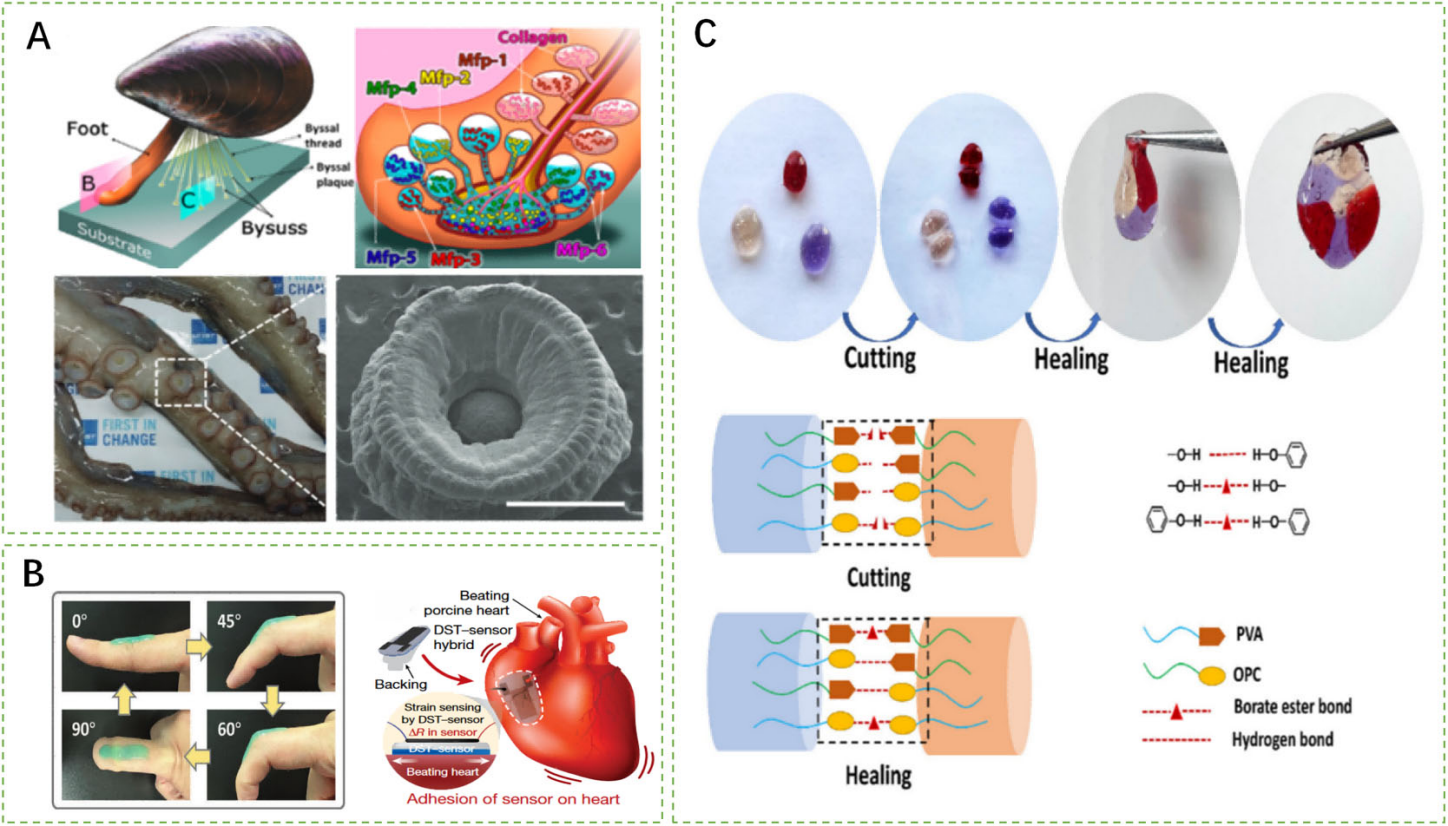
Schematic representation of research hotspots and challenges of smart responsive tissue adhesives. **(A)** Wet adhesion mechanism inspired by mussel foot protein and octopus suckers (112, 114). **(B)** Smart responsive tissue adhesives compliant with tissue elastic modulus (54, 115). **(C)** Mechanisms of self-healing characteristic of smart responsive tissue adhesives (72).

Current tissue adhesives possess multiple functions but cannot be applied in all environments for the reasons such as unstable adhesion in wet environments (112, 113), rupture of cross-linked networks under stress (114, 115), and non-compliance with the elastic modulus of the application site (16, 116). These unresolved issues limit the application of adhesives and hinder physiological activities. Researchers have proposed various solutions to address these problems (Figure 6).

### 4.1 Wet adhesion

Normal physiological activities and disease recovery processes require a large amount of tissue fluid, such as in the gastrointestinal environment, cardiovascular system, or wound healing process. However, excessive exudation of tissue fluid negatively impacts the adhesion strength of adhesives (117). Therefore, the wet adhesion capability of smart responsive tissue adhesives is crucial.

Inspired by mussel foot proteins, researchers have discovered that the catechol-based adhesion of dihydroxyphenylalanine (DOPA) enables high-strength adhesion in moist environments. Fan et al. developed a poly(acrylic acid-co-3-((8,11,13)-pentadeca-trienyl) benzene-1,2-diol (UCAT)-chitosan hydrogel (P(AA-co-UCAT)-CS) that interacts with -NH2 and -SH groups on tissues to maintain good adhesion even underwater (118). The adhesive retains an adhesion strength of 1200 J/m^2^ after soaking in water for 6 h. Furthermore, Zhao et al. formulated a thermally responsive adhesive coating using DOPA polymer, adamantine (AD), methoxyethyl acrylate (MEA) monomer, poly(N-isopropylacrylamide) (pNIPAM), and β-cyclodextrin (β-CD) (36). This adhesive coating achieves an adhesion strength of 4370 mJ/m^2^ to silicon substrates underwater.

The adhesion mechanisms and special structures of octopus suckers provide physical inspiration for wet adhesion (1). Inspired by the adhesive suction force generated by the deformation of suckers, Baik et al. proposed octopus-inspired architectures (OIAs) (66). Under preload, this structure achieves adhesion strengths of 41 and 154 kPa underwater and in silicone oil environments, respectively. Additionally, mimicking the protruding structures inside the suckers, Lee et al. designed OHA composed of pNIPAM (37). This adhesive absorbs water and swells at room temperature with its wet adhesion strength increasing as the effective adhesion area enlarges.

### 4.2 Self-healing properties

Hydrogel adhesives deform within the range of applied stress. However, when the stress exceeds this range, the crosslinked networks of adhesives collapse, and the integrity and mechanical properties are compromised (115). Self-healing properties could maintain adhesive integrity, preventing tissues or wounds from direct contact with the external environment to avoid contamination (114).

Smart tissue adhesives employ reversible chemical bonds to achieve the characteristic of self-healing, such as metal-ligand bonds, borate bonds, and hydrogen bonds, which congregate at incisions when the adhesives are cut (119). When the incised sides are approximated, complexation occurs, restoring the integrity of the adhesive. Xu et al. developed a CIPU: Cu^2+^ adhesive that takes advantage of the reformation of reversible Cu^2+^-ligand bonds (114). Moreover, the PBO hydrogel synthesized by He et al. realizes self-healing through reversible borate bonds and hydrogen bonds (72). With self-healing characteristics, smart tissue adhesives could maintain similar mechanical properties as before (113, 120).

### 4.3 Compliance with tissue elastic modulus

Different human tissues have varying elastic modulus. Adhesives with mismatched elastic modulus have a short lifespan, thereby limiting the normal physiological activities of tissues or organs (121). For tissue adhesives, appropriate elastic modulus is crucial and cannot be overlooked.

If the elastic modulus of cardiovascular adhesives does not match that of cardiovascular tissues, the adhesive is prone to detach or limit heart pumping functions during the contraction-relaxation cycles of hearts (122). He et al. developed a bilayer CPAMC/PCA hydrogel patch for cardiac applications, which not only exhibits excellent on-demand adhesion but also has a suitable elastic modulus that ensures that the adhesive performs its intended functions without affecting the heart’s normal activities (56).

Peripheral vessels are exposed to high-pressure blood flow, and vascular sealing adhesives must possess sufficient burst pressure thresholds to ensure normal blood flow without the patch deforming or rupturing (123). Borden et al. developed an electroadhesion QDM gel with a burst pressure reaching up to 252 mmHg higher than human blood pressure (76). Although the burst pressure decreases with increased vessel rupture size, it can be enhanced by introducing multiple gel layers.

Additionally, different tissues have different requirements for adhesive elastic modulus, such as joints and other flexible areas. Li and Liu et al. created a D. lapponicus-inspired adhesion structure (DIAS) capable of providing stable adhesive strength in various environments (65). Its excellent extensibility ensures a secure attachment during repeated flexion-extension cycles at the wrist.

### 4.4 Biocompatibility and safety

Thermosensitive adhesives typically operate within biologically safe temperature ranges. While formulations containing freely released copper or iron ions may induce cytotoxicity through oxidative stress, strategic material design-such as controlling ion release and employing biocomplexes-can effectively mitigate these risks while maintaining antibacterial efficacy.

Current tissue adhesives face clinical challenges such as insufficient wet adhesion, weak mechanical toughness, and potential biocompatibility issues, which may lead to adhesion failure or inflammatory responses. From a regulatory perspective, their classification as Class III medical devices demands rigorous evidence of safety and efficacy, while the lack of standardized evaluation protocols further prolongs the approval process.

## 5 Conclusion

Smart tissue adhesives not only retain the characteristics of convenience, ease of use, and low technical requirements of traditional tissue adhesives but also innovatively demonstrate advantages such as reversible adhesion, minimally invasive detachment, and matched degradation rates. Compared to conventional adhesives, smart responsive tissue adhesives better align with the demands of biomedical applications. This review introduced the reversible adhesion mechanisms, biomedical application scenarios, and recent research hotspots of smart tissue adhesives. With opportunities come challenges, and it is believed that with the advancement of biomedical and materials science, multifunctional smart responsive tissue adhesive systems will become more sophisticated, find wider applications, and ultimately benefit clinical practice.
